# Combined Use of Shear Wave Elastography, Microvascular Doppler Ultrasound Technique, and BI-RADS for the Differentiation of Benign and Malignant Breast Masses

**DOI:** 10.3389/fonc.2022.906501

**Published:** 2022-05-24

**Authors:** Bin Wang, Yu-Yuan Chen, Si Yang, Zhen-Wen Chen, Jia Luo, Xin-Wu Cui, Christoph F. Dietrich, Ai-jiao Yi

**Affiliations:** ^1^ Department of Medical Ultrasound, Yueyang Central Hospital, Yueyang, China; ^2^ Department of Medical Ultrasound, Tongji Hospital, Tongji Medical College, Huazhong University of Science and Technology, Wuhan, China; ^3^ Department Allgemeine Innere Medizin, Kliniken Hirslanden Beau Site, Salem und Permanence, Bern, Switzerland

**Keywords:** breast mass, ultrasound, shear wave elastography, Angio PLUS, Breast Imaging Reporting and Data System

## Abstract

**Objective:**

To evaluate the value of the combined use of Breast Imaging Reporting and Data System (BI-RADS), qualitative shear wave elastography (SWE), and AngioPLUS microvascular Doppler ultrasound technique (AP) for distinguishing benign and malignant breast masses.

**Materials and Methods:**

A total of 210 pathologically confirmed breast lesions in 210 patients were reviewed using BI-RADS, qualitative SWE, and AP. The sensitivity, specificity, negative predictive value (NPV), positive predictive value (PPV), accuracy, and area under the receiver operating characteristic curve (AUC) of BI-RADS and the combination of qualitative SWE and/or AP with BI-RADS were compared, respectively.

**Results:**

Compared with using BI-RADS alone, the use of combined qualitative SWE and/or AP with BI-RADS had higher AUC values (*P* < 0.001). Besides this, the combination of qualitative SWE and AP with BI-RADS had the best diagnostic performance for differentiating between benign and malignant masses. When AP and SWE were combined with BI-RADS, 49/76 benign masses were downgraded from BI-RADS category 4a into BI-RADS category 3, while no benign masses were upgraded from BI-RADS category 3 into BI-RADS category 4a. Three sub-centimeter malignant masses were downgraded from BI-RADS category 4a into BI-RADS category 3, while three malignant masses remain in BI-RADS category 3 due to a benign manifestation in both AP and qualitative SWE. Moreover, 5/6 of them were sub-centimeter masses, and 4/6 of them were intraductal carcinoma. The sensitivity, specificity, PPV, NPV, accuracy, and AUC were 91.0%, 81.1%, 69.3%, 95.1%, 84.3%, and 0.861 (95% confidence interval, 0.806–0.916; *P* < 0.001), respectively. Compared with BI-RADS alone, the sensitivity slightly decreased, while the specificity, PPV, NPV, and accuracy were significantly improved.

**Conclusion:**

Combination of qualitative SWE and AP with BI-RADS improved the diagnostic performance in differentiating benign from malignant breast lesions, which is helpful for avoiding unnecessary biopsies. However, we should be careful about the downgrading of sub-centimeter BI-RADS 4a category lesions.

## Introduction

Breast cancer is the leading cause in the morbidity and mortality of women all over the world. In recent years, the incidence of breast cancer has been increasing (1, 2). Breast ultrasound gives real-time results, is convenient, is of a low cost, and is non-radiative; thus, it has been a crucial tool for screening of breast lesions. The fifth edition of the Breast Imaging Reporting and Data System (BI-RADS) was widely used to standardize the risk evaluation of breast lesions (3). However, there was a highly variable rate of breast cancer and a high rate of benign lesions (61.2%) (4) in BI-RADS category 4, which might cause unnecessary biopsies.

Some studies showed that breast cancer was highly related to the angiogenesis of microvessels (5). Microvessels are essential to the growth, invasion, and survival of breast tumors. Furthermore, the microvascular architecture in benign tumors was markedly different from that of the malignant ones (6). Currently, color Doppler flow imaging (CDFI), power Doppler imaging (PDI), contrast-enhanced ultrasound (CEUS), and dynamic contrast-enhanced magnetic resonance imaging (CE-MRI) are widely used to detect vascularity in breast tumors (7–9). CDFI is the most widely used method, which is noninvasive and simple to operate, and it can provide some vascularity characteristics which suggest malignancy, such as hypervascularity, central or penetrating vessels, and a branching or disordered morphology (5, 10, 11). Compared with CDFI, PDI has an advantage in the detection of low-velocity vessels and allows one to observe blood vessels in real time. However, CDFI was limited in evaluating vessels <0.2 mm in diameter, PDI has low sensitivity in the detection of microvessels, and the differences of vascularity between malignant and benign lesions had great overlaps (12, 13), which impacted their differentiation ability compared with other invasive methods, such as CEUS and CE-MRI (14).

Angio PLUS microvascular Doppler ultrasound technique (AP) is an innovative Doppler ultrasound technique (Aixplorer, Supersonic Imaging, France). AP relies on two key pillars to achieve unfocused or plane waves and 3D wall filtering. Plane or unfocused waves are sent into the body at the maximum allowed pulse repetition, and all pixels of the explored tissue can be reconstructed from a single unfocused insonification with a significantly higher sampling rate than in classical CDFI. Thus, AP can increase the imaging sensitivity and resolution to get a better detection of microvessels (15, 16).

Shear wave elastography (SWE) is a technique that can assess tissue stiffness by using acoustic radiation to induce mechanical vibration. The SWE images are displayed in a real-time color overlay box with different colors to indicate the speed of the shear wave (in meters per second, m/s) or the degree of tissue stiffness (Young’s modulus; in kilopascal, kPa) in each pixel. The stiffness of a tissue can be assessed by a quantitative measurement or a qualitative map. Previous studies have found that a quantitative measurement or a qualitative map is useful in the diagnosis of breast lesions, and it had been proven to be a reproducible technique (17–19).

To our best knowledge, there was no study on the combined use of SWE and AP with BI-RADS for the differentiation of benign and malignant breast masses. The purpose of this study was to compare the diagnostic efficiency of AP, SWE, and the combined use of SWE and/or AP with BI-RADS for breast lesions. We attempt to find optimal methods to differentiate benign breast lesions from malignant ones.

## Materials and Methods

This prospective study was approved by the ethics committee of our institution.

### Patients

From August 2018 to July 2019, a total of 210 patients with solid breast masses were recruited. The inclusion criteria were patients aged 18 years or older with at least a solid mass detected on the B-mode ultrasound, and the pathology of all the breast masses was confirmed *via* ultrasound-guided core needle biopsy and/or surgery in 1 month according to standard clinical protocols. People were excluded if lactating or pregnant, had a previous needle biopsy, or had any treatment of the same lesions. When one patient had multiple lesions, the most suspicious lesion was included.

### Ultrasound Examination

All ultrasound examinations, including grayscale ultrasound, CDFI, AP, and SWE were performed with a high-frequency transducer (L15-4 or L10-5 Aixplorer, Supersonic Imaging, France).

Bilateral breast ultrasound was performed. When a target lesion was detected, the general characteristics were observed. The B-mode features of the lesion were clearly depicted, including the shape, margin, orientation, echo pattern, posterior features, calcifications, and associated features. Each lesion was classified as either category 3 (probably benign), 4a (low suspicion for malignancy), 4b (moderate suspicion for malignancy), 4c (high suspicion for malignancy), or 5 (highly suggestive of malignancy) according to the fifth edition of BI-RADS (3).

Two orthogonal planes containing the richest vascularity of each lesion were scanned with CDFI and AP. The following settings were used for the CDFI examination: the color velocity scale was adjusted at 3 cm/s, and the color gain was adjusted adequately as the background noise was just suppressed. The region of interest included the whole masses and the breast tissue surrounding the lesion for at least 3 mm. For those large breast masses (>40 mm) that were not single-screen included, we observed the lesions in different planes to cover all the masses and the surrounding areas.

In terms of the setting for AP, gain settings were tuned to the optimum degree without color noise for each imaging. During the examination of CDFI and AP, the patients were asked to hold their breath for a while, and no pressure was applied through the transducer to prevent the vessels from collapsing (12).

After the AP imaging, SWE imaging was obtained by the following recommendations: SWE imaging examination was induced by the transducer without pressure. The region of interest included the whole lesion and breast tissue surrounding the lesion for at least 3 mm. The stiffness range of the color map was from blue to red (0–180 kPa). The standard SWE imaging was obtained with several seconds of immobilization.

All sonographic scanning was performed by the same investigator who had more than 20 years of experience in breast ultrasound and 2 years of experience in AP and SWE.

### Imaging Analysis

The imaging data were analyzed by two radiologists (about 10 years of experience in breast ultrasound and 2 years of experience in AP and SWE) who were blinded to the pathological results. The third investigator evaluated the lesions when a disagreement occurred. Pathology was considered to be a golden standard.

**Figure 1:**
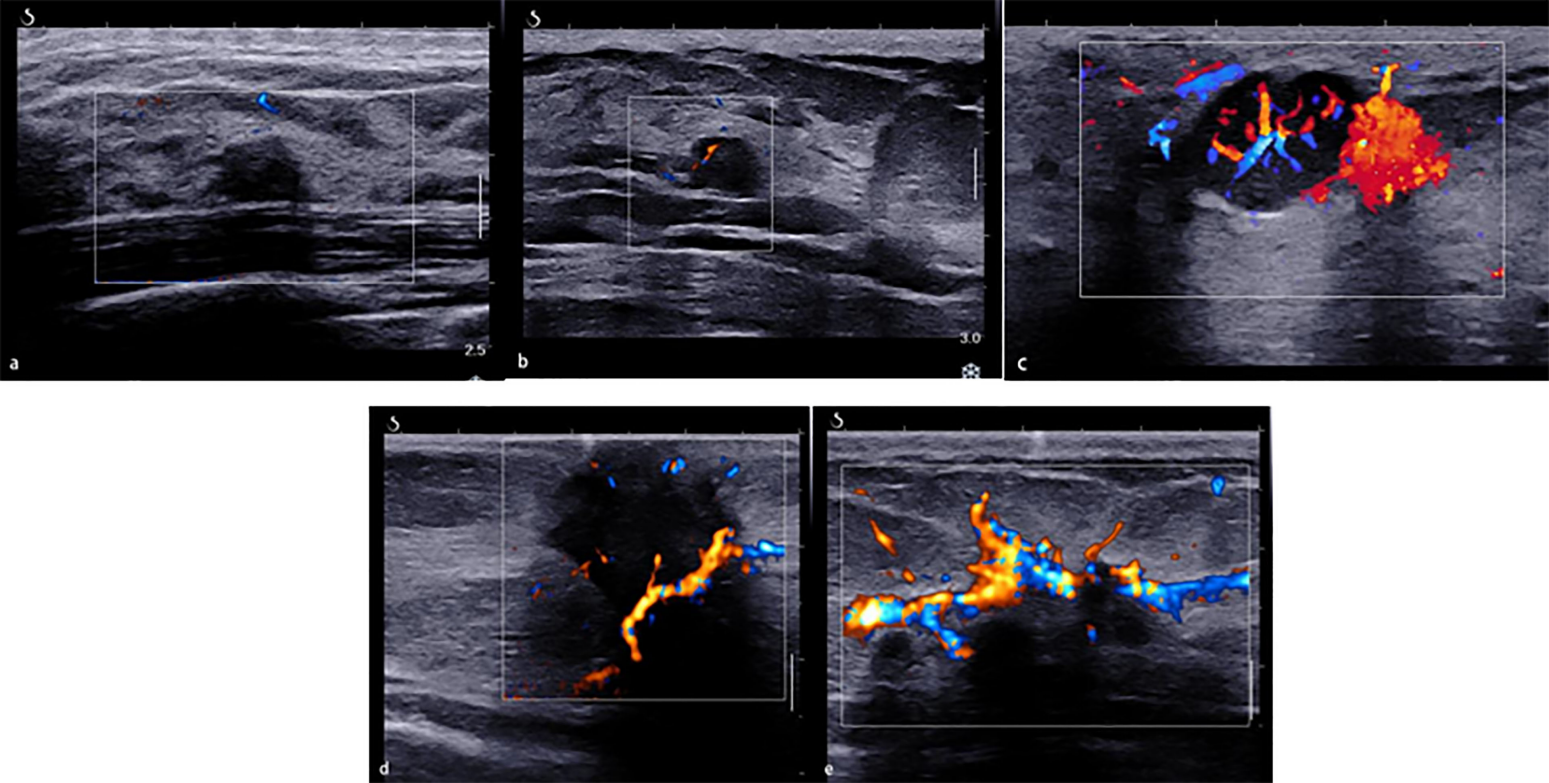
The morphologic and distribution features of Angio PLUS. **(A)** Non-vascular pattern. **(B)** A linear or curvilinear pattern. **(C)** A treelike pattern. **(D)** A root hair-like pattern. **(E)** A crab claw-like pattern.

The morphologic and distribution features detected on AP were divided into five patterns according to the shape of the vascular networks: (1) non-vascular pattern, which was due to a lack of vessels; (2) a linear or curvilinear pattern, with a single or a few straight or slightly curved vessels without crossing which were found inside the lesion; (3) a treelike pattern, which consisted of proportioned microvessels branching within the lesions; (4) a root hair-like pattern, which was dominated by a twist and a chaotic arrangement, and the irregular vessels within the lesions had less than two enlarged and twisted vessels surrounding the lesion; and (5) a crab claw-like pattern, which was characterized by radial vessels and with small speculated vessels commonly seen in the peripheral region (**Figure 1**).

**Figure 2:**
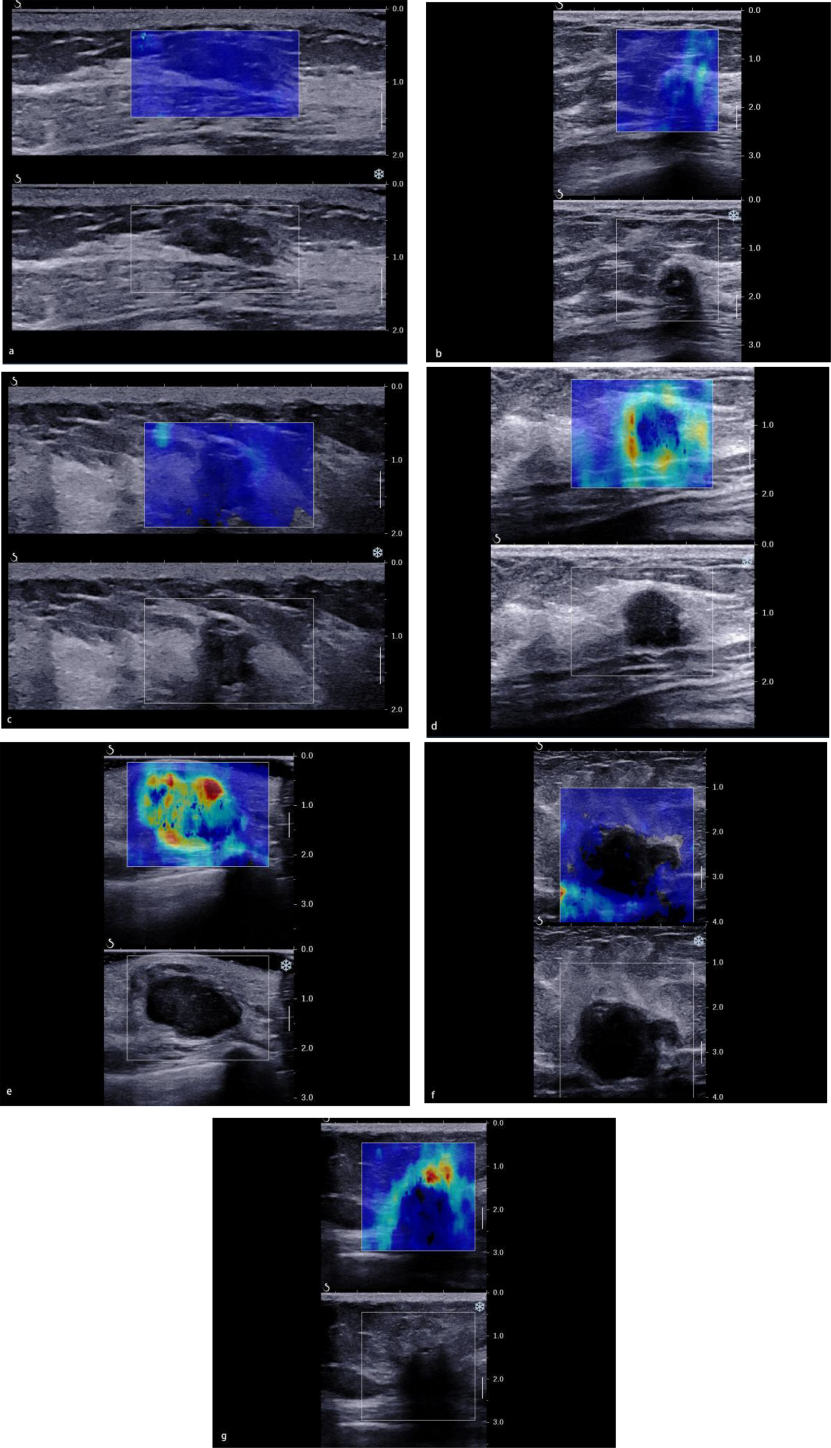
The color pattern features of shear wave elastography. **(A)** No finding. **(B)** Vertical stripes pattern. **(C)** Spots pattern. **(D)** Rim of stiffness pattern. **(E)** Colored lesion pattern. **(F)** Void center pattern. **(G)** Horseshoe pattern.

As for the qualitative SWE features, we used seven color patterns in this study (20): (1) no finding: no difference is observed at the margin of or inside the lesion with the color around the lesion (homogeneously blue); (2) vertical stripes pattern: a color is observed at the margin of or inside the lesion, which differs from the color around the lesion. The differing color extends beyond the lesion and continues vertically in cords on the cutaneous side and/or the thoracic wall; (3) spots pattern: colored areas are visible above and/or below the lesion; and (4) rim of stiffness pattern: a localized colored area appears at the margin of the lesion and creates a continuous closed circle; (5) colored lesion pattern: colored areas are heterogeneously visible inside the lesion; (6) void center pattern: There is a lack of SWE signal inside the lesion. The rest of the SWE Box fills correctly; and (7) horseshoe pattern: a localized colored area appears at the margin of the lesion and creates an open circle (**Figure 2**).

### Statistical Analysis

According to the final pathological results, the sensitivity, specificity, positive predictive value (PPV), negative predictive value (NPV), accuracy, and area under the receiver operating characteristic curve (AUC) of BI-RADS and the combination of BI-RADS, qualitative SWE, and AP were calculated and compared, respectively. A *P*-value <0.05 was considered statistically significant. SPSS 22.0 was used for all statistical analysis.

## Results

### Pathological Findings

**Figure 3:**
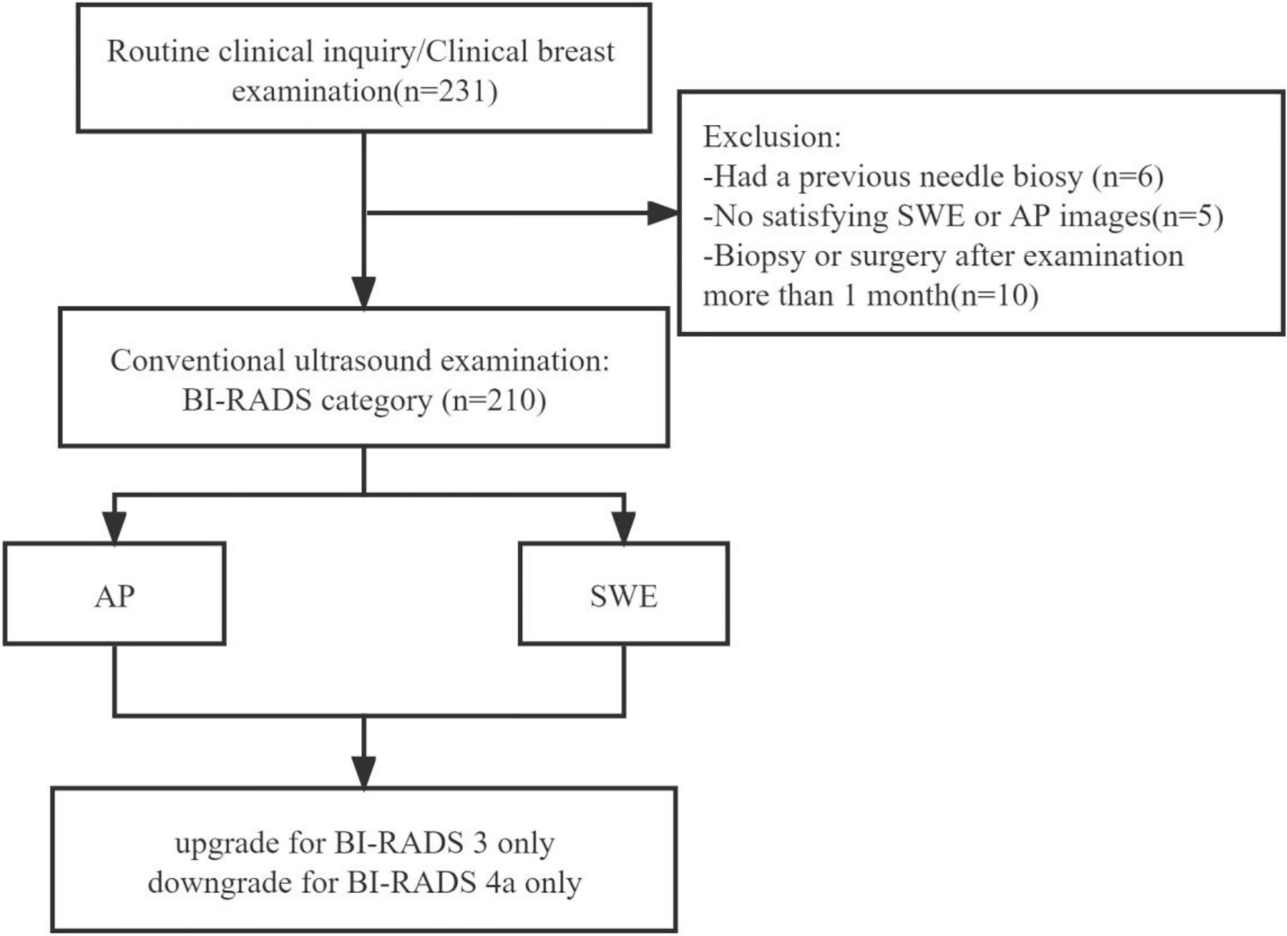
Flow chart for the selection of breast lesions.

Of the 210 enrolled breast lesion cases (**Figure 3**), histopathologically, 67 (67/210) lesions were malignant, and 143 (143/210) lesions were benign (**Table 1**). Finally, 70 (33.3%) lesions were assigned to BI-RADS category 3, of which 3 (4.3%) were malignant. A total of 140 lesions were assigned to BI-RADS categories 4 and 5 (4a, 68 lesions; 4b, 32 lesions; 4c, 35 lesions; and 5, 5 lesions), and the confirmed malignant rates for 4a, 4b, 4c, and 5 were 10.3, 56, 97, and 100%, respectively.

**Table 1:** Pathology of 210 breast lesions.

Pathology result	Number of lesions
Benign	143
Fibroadenoma	98
Fibrocystic mastopathy	29
Benign phyllodes tumor	2
Mastitis	9
Breast abscess	3
Intraductal papilloma	2
Malignant	67
Invasive ductal carcinoma	47
Intraductal carcinoma	11
Invasive lobular carcinoma	5
Papillary carcinoma	2
Mucinous carcinoma	2

### Reclassification for BI-RADS Category 3 and 4a Lesions

In this study, the modified BI-RADS category combining SWE or/and AP with ultrasound (US) was only for BI-RADS categories 3 and 4a. When combining AP with BI-RADS, the morphologic and distribution features of BI-RADS category 4a manifested a non-vascular pattern, a linear or curvilinear pattern, and a treelike pattern which were downgraded into BI-RADS category 3. The morphologic and distribution features of BI-RADS category 3, which manifested a root hair-like pattern and a claw-like pattern, were upgraded into BI-RADS category 4a. When combining SWE with BI-RADS alone, the qualitative SWE features of BI-RADS category 4a showed no finding, vertical stripes, and spots above/below, which were downgraded to BI-RADS category 3. The qualitative SWE features of BI-RADS category 3 showed rim of stiffness, horse shoe, void center, and colored lesion, which were upgraded to BI-RADS 4a category (**Table 2**). When combining SWE and AP with BI-RADS, BI-RADS 4a category was downgraded into BI-RADS 3 category with the morphologic and distribution features that manifested a non-vascular pattern, linear or curvilinear pattern, and treelike pattern, and the qualitative SWE features showed no finding, vertical stripes, and spots above/below, while BI-RADS 3 category was upgraded into BI-RADS 4a category with the morphologic and distribution features manifested as root hair-like pattern and claw-like pattern, and the qualitative SWE features showed rim of stiffness, horse shoe, void center, and colored lesion.

**Table 2:** Comparison of qualitative SWE and AP features between benign and malignant lesions with pathological results.

	Benign	Malignant	*P*
Age^a^	44 (37–49)	53 (47–49)	<0.001
Size (mm)^a^	12 (9–18)	20 (13–28)	<0.001
AP^b^			<0.001
Non-vascular pattern	44	4	
Linear or curvilinear pattern	73	19	
Treelike pattern	9	1	
Root hair-like pattern	10	13	
Crab claw-like pattern	7	30	
SWE^b^			<0.001
No finding	84	9	
Vertical stripes pattern	15	1	
Spots pattern	14	1	
Rim of stiffness pattern	21	31	
Colored lesion pattern	6	15	
Void center pattern	1	3	
Horseshoe pattern	2	7	

a Data are expressed as median (interquartile range).

b Data are expressed as numbers.

AP, Angio PLaneWave UltraSensitive ultrasound imaging; SWE, shear wave elastography.

### Diagnostic Performance of BI-RADS, BI-RADS + AP, and BI-RADS + SWE and the Combination of SWE and AP With BI-RADS

**Figure 4:**
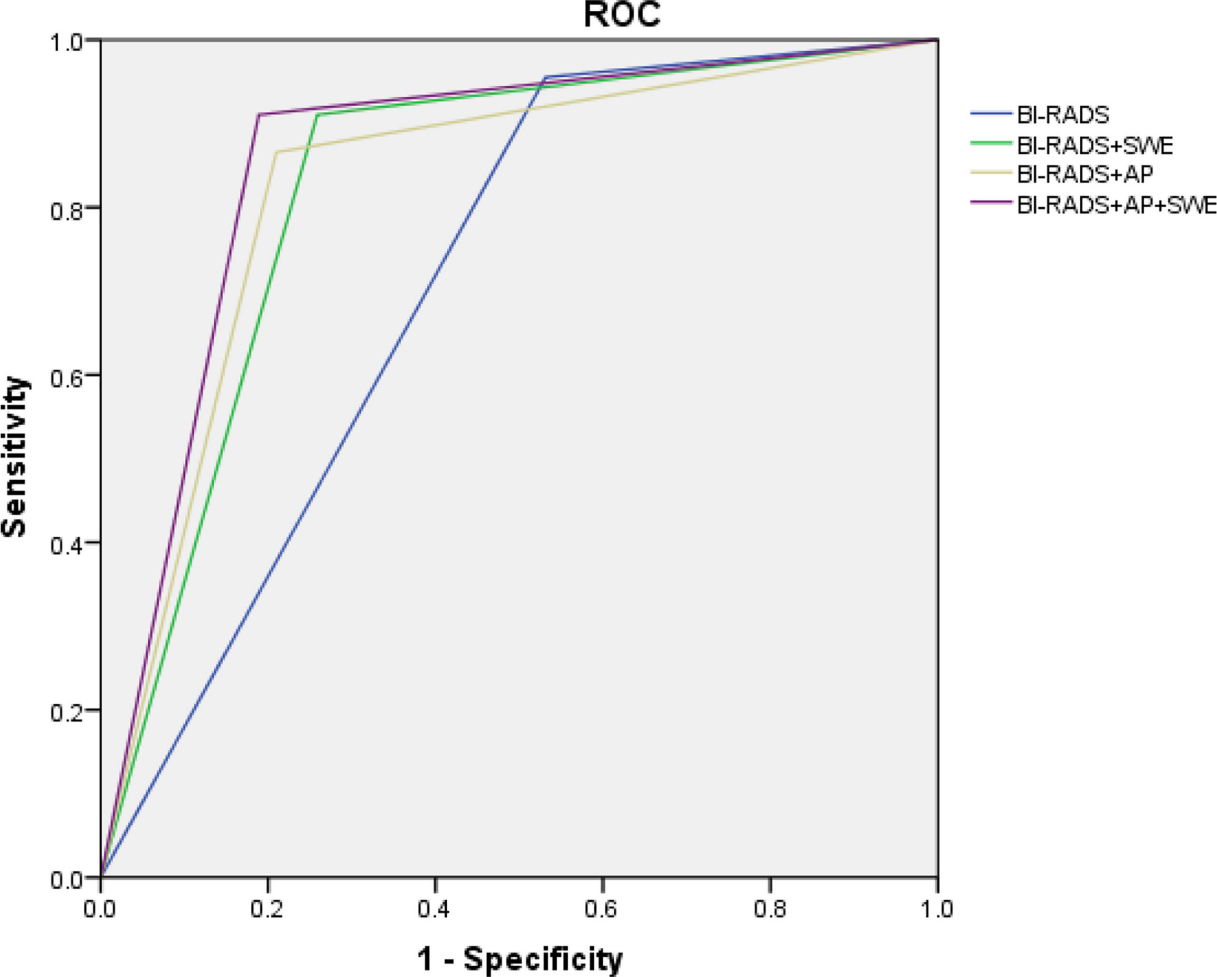
ROC of Breast Imaging Reporting and Data System alone and combined qualitative shear wave elastography and/or AngioPLUS microvascular Doppler ultrasound technique. ROC, receiver operating characteristic; AUC, area under the ROC curve.

BI-RADS category 3 was regarded as benign, while BI-RADS categories 4a, 4b, 4c, and 5 were regarded as malignant. The AUC of B-mode US with BI-RADS was 0.712 [95% confidence interval (CI), 0.643–0.781]. The sensitivity, specificity, PPV, NPV, and accuracy were 95.5, 46.9, 45.7, 95.7, and 62.4%, respectively. The sensitivity, specificity, PPV, NPV, and accuracy of AP alone were 59.7, 87.4, 69.0, 82.2, and 78.6%, respectively. The sensitivity, specificity, PPV, NPV, and accuracy of SWE alone were 83.6, 79, 65.1, 91.1, and 80.5%, respectively (**Table 3**). When AP was combined with BI-RADS, 89.7% (61/68) of BI-RADS 4a lesions were downgraded into BI-RADS 3, including 9.8% (6/61) malignant lesions. The AUC of BI-RADS and AP was 0.828 (95% CI, 0.767–0.889). The sensitivity, specificity, PPV, NPV, and accuracy were 86.6, 79.0, 65.9, 92.6, and 81.4%, respectively. When SWE was combined with BI-RADS, the sensitivity was reduced from 95.5 to 91%, while the specificity increased from 46.9 to 74.1%. Four malignant breast masses were downgraded from BI-RADS category 4a into BI-RADS 3 category, and one malignant breast mass was upgraded from BI-RADS category 3 into BI-RADS category 4a. The AUC of BI-RADS and SWE was 0.826 (95% CI, 0.767–0.885). The sensitivity, specificity, PPV, NPV, and accuracy were 91.0, 74.1, 62.2, 94.6, and 79.5%, respectively. When AP and SWE were combined with BI-RADS, 49/76 (64.5%) benign masses were downgraded into BI-RADS category 3, while no benign masses were upgraded from BI-RADS category 3 into BI-RADS category 4a. Three sub-centimeter malignant masses were downgraded into BI-RADS category 3, and three malignant masses remain in BI-RADS category 3 due to benign manifestation in both AP and qualitative SWE—5/6 of them were sub-centimeter masses, and 4/6 of them were intraductal carcinoma. Compared with BI-RADS alone, the diagnostic performance of the combination of AP and qualitative SWE improved. The AUC was increased from 0.712 (95% CI, 0.643–0.781) to 0.861 (95% CI, 0.806–0.916) (*P* < 0.001) (**Figure 4**), and the sensitivity slightly decreased, while the specificity, PPV, NPV, and accuracy were increased from 46.9, 45.7, and 62.4% to 81.1, 69.3, and 84.3%, respectively (**Table 4**).

**Table 3:** Diagnostic performance of Breast Imaging Reporting and Data System, shear wave elastography, and AngioPLUS microvascular Doppler ultrasound technique in distinguishing malignant from benign lesions.

	Sensitivity (%)	Specificity (%)	Positive predictive value (%)	Negative predictive value (%)	Accuracy (%)
BI-RADS	95.5	46.9	45.7	95.7	62.4
AP	59.7	87.4	69	82.2	78.6
SWE	83.6	79.0	65.1	91.1	80.5

**Table 4:** Diagnostic performance of the combined qualitative parameters of shear wave elastography (SWE) or/and AngioPLUS microvascular Doppler ultrasound technique (AP) with Breast Imaging Reporting and Data System (BI-RADS) in distinguishing malignant from benign lesions.

	Sensitivity (%)	Specificity (%)	Positive predictive value (%)	Negative predictive value (%)	Accuracy (%)	Area under the receiver operating characteristic curve (95%CI)	*p* ^a^
BI-RADS	95.5 (64/67)	46.9	45.7	95.7	62.4	71.2 (0.643–0.781)	–
BI-RADS + AP	86.6 (58/67)	79	65.9	92.6	81.4	82.8 (0.767–0.889)	<0.001
BI-RADS + SWE	91.0 (61/67)	74.1	62.2	94.6	79.5	82.6 (0.767–0.885)	<0.001
BI-RADS + AP + SWE	91.0 (61/67)	81.1	69.3	95.1	84.3	86.1 (0.806–0.916)	<0.001

Data are expressed as percentage (numbers).

a Comparison of the diagnostic performance between BI-RADS alone with the combination of the qualitative SWE and/or AP with BI-RADS.

## Discussion

Our studies found that qualitative SWE can provide extra stiffness information of breast masses, AP can depict the morphologic and distribution features of microvessels, and the combination of qualitative SWE and AP with BI-RADS could significantly improve the diagnostic specificity to avoid unnecessary biopsy.

The American College of Radiology (ACR) BI-RADS lexicon mainly focused on morphology, which is widely used in ultrasound examination. This system could improve the reproducibility and reliability of cancer risk assessment (21, 22), and it has high sensitivity but with a low PPV with a substantial number of false-positive findings that cause unnecessary biopsies, which is the major limitation (13, 14). In this study, the sensitivity and specificity of using BI-RADS alone were 95.5 and 46.9%, respectively.

According to the 2013 ACR BI-RADS guideline, vascularity is one of the associated features. It is classified into 3 types in CDFI or PDI, including absent, internal vascularity, and vessels in rim. However, angiogenesis plays a critical role in tumor development and metastasis. Therefore, it is important to use vascularity as a diagnostic feature. However, microvessel detection was limited in CDFI or PDI, and AP can display more internal small vessels.

There were a few studies on morphologic and distribution features in differentiating benign breast lesions from malignant ones. Feng et al. (23) found that breast lesions with a centrally distributed branching or chaotic vessels were informative signs of breast malignancy. Chang et al. (24) found morphologic and tortuous features of microvessels in 3D power Doppler ultrasound, which was useful in distinguishing benign from malignant lesions. Xiao et al. (25) found that malignant lesions always showed penetrating vessels and spiculated or radial vessels in the peripheral regions, displaying root hair-like or crab claw-like patterns, whereas benign lesions mainly showed peripheral annular, non-vascular, linear, and treelike patterns.

In this study, we observed the morphologic and distribution features of AP in breast lesions. It was consistent with Xiao’s study, but we found that there was an overlap between benign and malignant breast lesions, especially between inflammatory lesions and malignant breast lesions. Furthermore, since vascularity is an important factor for tumor differentiation, and AP performed well in vascular detection, in our study, we tried to combine AP with BI-RADS. When the morphologic and distribution features in AP were used alone, the sensitivity was reduced from 95.5 to 59.7%, while the specificity increased from 46.9 to 87.4%. Furthermore, 40.3% (27/67) malignant lesions were manifested in a non-vascular or linear pattern. It may be because the vascular velocity of some microvessels was lower than the threshold for AP, and some ductal carcinoma *in situ* or invasive cancers manifested low blood perfusion (26), which also indicates that the angiogenesis in malignant breast tumors was heterogeneous (27).

When AP was combined with BI-RADS, 6/61 malignant lesions were missed. All these 6 malignant lesions manifested a non-vascular or linear pattern. The final pathological results of these lesions were different grades of intraductal carcinoma or mucinous carcinoma: two were high-grade intraductal carcinoma, two were intermediate-grade intraductal carcinoma, one was low-grade intraductal carcinoma, which may be related to that part of intraductal carcinomas where there is a lack of blood supply, and two intraductal carcinoma were located at a depth of over 20 mm. The maximal diameter of one lesion was 5 mm, so AP could not depict a complete vascular caliber. What is more, one benign lesion was upgraded from BI-RADS 3 to BI-RADS 4a category because the morphologic and distribution features of AP manifested a claw-like pattern. Therefore, it was useful but insufficient to consider morphologic and distribution features as the only diagnostic feature in the interpretation.

In recent years, there were many studies on the differentiation of benign and malignant breast lesions with elastography. Lin et al. (20) found that malignant breast lesions mainly manifested rim of stiffness pattern, colored lesion pattern, void center pattern, and horseshoe pattern with qualitative SWE, while benign breast lesions mainly manifested no finding, vertical stripes pattern, and spots pattern, which were consistent with our study.

When SWE was combined with BI-RADS, four malignant breast lesions were missed. All these masses showed no finding with qualitative SWE, and 2/4 of these lesions were high-grade intraductal carcinoma, 1/4 of these was intermediate-grade intraductal carcinoma, 1/4 of these was invasive ductal carcinoma, and 3/4 of these lesions were sub-centimeter masses. Intraductal carcinoma is a precancerous lesion with a complicated pathological entity, so the lack of morphologic changes was difficult to be detected by ultrasound (28), and the stiffness may be lower than that of invasive carcinoma, which caused the no-finding manifestation of qualitative SWE. In addition, 1 malignant breast mass manifested a horseshoe pattern and was upgraded into BI-RADS category 4a. The final pathology result was invasive ductal carcinoma, and the maximum diameter of the lesion was 9 mm. Therefore, SWE features were useful for differentiation between benign and malignant lesions, and compared with AP, 4 sub-centimeter malignant lesions manifested malignant qualitative SWE features earlier than the malignant morphologic and distribution features of AP. However, 4 malignant breast lesions were downgraded into BI-RADS category 3, which was recommended with a short-time follow-up. Thus, it is insufficient to use SWE alone to differentiate benign from malignant breast lesions.

**Figure 5:**
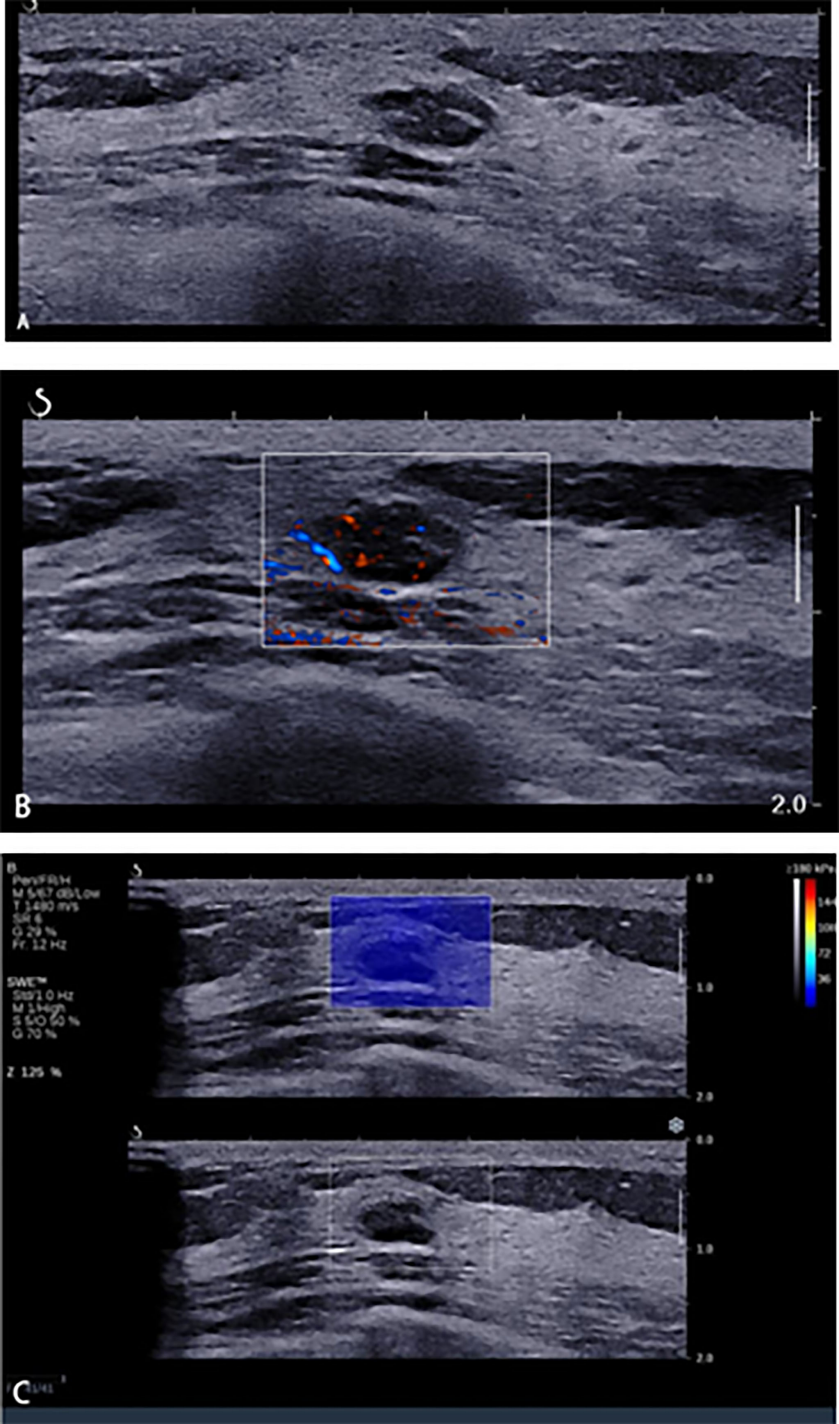
A 45-year-old woman with breast lesions. **(A)** Conventional B-mode ultrasound revealed a 10 × 6-mm round, hypoechoic lesion with a clear margin in the right breast, which was categorized as Breast Imaging Reporting and Data System (BI-RADS) 4a. **(B)** The morphologic and distribution features of the microvessels in AngioPLUS microvascular Doppler ultrasound technique (AP) followed a linear pattern. **(C)** The qualitative shear wave elastography (SWE) feature showed no finding. Considering the benign manifestation both in AP and qualitative SWE, the final category was downgraded into BI-RADS 3. The ultrasound-guided biopsy revealed the lesion as fibroadenoma.

**Figure 6:**
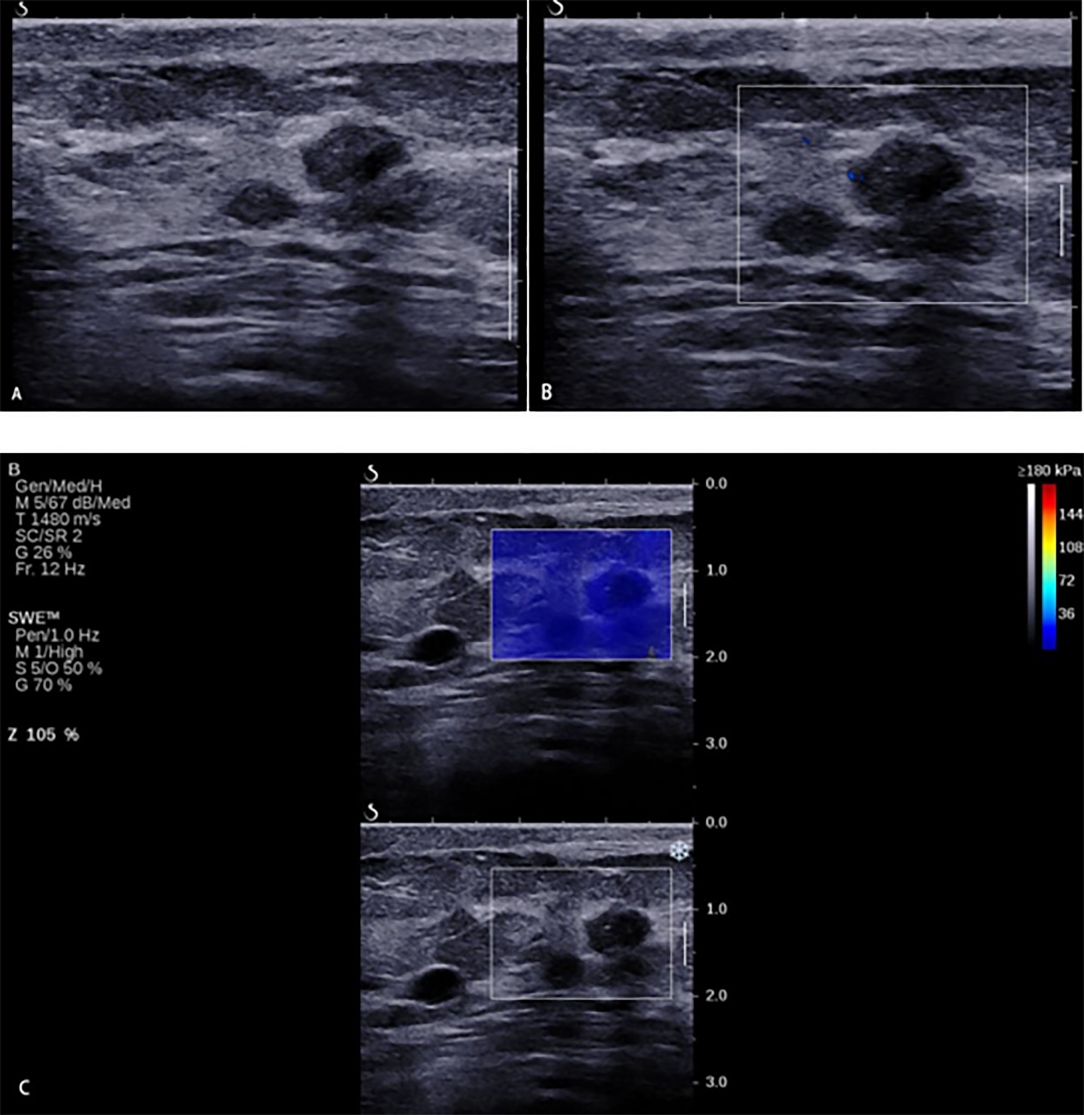
A 33-year-old woman with breast lesions. **(A)** The conventional B-mode ultrasound revealed an 8 × 7-mm round, hypoechoic lesion with unclear margin in the right breast, which was categorized as Breast Imaging Reporting and Data System (BI-RADS) 4a. **(B)** The morphologic and distribution features of the microvessels in AngioPLUS microvascular Doppler ultrasound technique followed a linear pattern. **(C)** The qualitative shear wave elastography (SWE) feature showed no finding. Considering the benign manifestation both in AP and qualitative SWE, the final category was downgraded into BI-RADS 3. The ultrasound-guided biopsy revealed the lesion as intermediate-grade intraductal carcinoma.

There was a study (29) which reported that the combination of the quantitative values of SWE and the vascular index in SMI could significantly improve the accuracy and specificity, but the sensitivity decreased slightly, which was consistent with this study. However, the ultrasound system of a previous study is different with this study, and the cutoff values of quantitative SWE and vascular index for differentiating benign from malignant breast masses may be varied in different ultrasound systems. The SWE technique of this study had been widely used and recognized in official clinical guidelines (20, 30). This study first found that the combination of qualitative SWE and AP with BI-RADS had an added value. When we combined SWE and AP with BI-RADS to modify the original BI-RADS category, we found that 64.5% of benign BI-RADS category 4a masses were downgraded into BI-RADS category 3 (**Figure 5**). The diagnostic specificity was significantly improved, thus avoiding a lot of unnecessary biopsies. However, 6 malignant masses were misdiagnosed due to benign manifestation in both AP and qualitative SWE; 5/6 of them were sub-centimeter, and 4/6 of them were intraductal carcinoma (**Figure 6**). For some sub-centimeter intraductal carcinoma masses, AP and SWE cannot detect obvious malignant features, which may be caused by heterogeneous angiogenesis or a small collagen fiber area. Thus, we should be careful in downgrading sub-centimeter BI-RADS category 4a lesions, and especially for sub-centimeter intraductal carcinoma masses, mammography and MRI can be combined if necessary.

There were several limitations in this study. First, it was a preliminary study in one center with a small sample. Second, some final pathology results of the patients were not available, which may have caused a selection bias of enrollment. Third, the time span was short, and the pathological categories were limited.

## Conclusion

In conclusion, the morphologic and distribution features of microvessels in AP and the stiffness information in SWE were useful in the differential diagnosis of benign and malignant lesions. The combination of qualitative SWE and AP with BI-RADS could improve specificity, thus avoiding unnecessary biopsy. However, we should be careful when downgrading sub-centimeter BI-RADS category 4a lesions.

## Data Availability Statement

The datasets presented in this article are not readily available because the raw dataset contain the information of the enrolled patients. Requests to access the datasets should be directed to Xin-wu Cui (cuixinwu@live.cn).

## Ethics Statement

The studies involving human participants were reviewed and approved by the ethics committee of Yueyang Central Hospital. The patients/participants provided their written informed consent to participate in this study.

## Author Contributions

Conception and design: BW, AY, X-WC, and CD. Drafting of the article: BW, Y-YC, and SY. Critical revision of the article for important intellectual content: BW, JL, and Z-WC. All authors contributed to the article and approved the submitted version.

## Funding

This work was supported by the National Natural Science Foundation of China (numbers 82071953 and 21878237) and the Science and Technology Department of Hunan Province (number 2020SK52705).

## Conflict of Interest

The authors declare that the research was conducted in the absence of any commercial or financial relationships that could be construed as a potential conflict of interest.

## Publisher’s Note

All claims expressed in this article are solely those of the authors and do not necessarily represent those of their affiliated organizations, or those of the publisher, the editors and the reviewers. Any product that may be evaluated in this article, or claim that may be made by its manufacturer, is not guaranteed or endorsed by the publisher.
